# Sulforaphane promotes *C. elegans* longevity and healthspan via DAF-16/DAF-2 insulin/IGF-1 signaling

**DOI:** 10.18632/aging.202512

**Published:** 2021-01-20

**Authors:** Zhimin Qi, Huihui Ji, Monika Le, Hanmei Li, Angela Wieland, Sonja Bauer, Li Liu, Michael Wink, Ingrid Herr

**Affiliations:** 1 Molecular OncoSurgery, Section Surgical Research, Department of General, Visceral and Transplant Surgery, University of Heidelberg, Heidelberg, Germany; 2 Institute of Pharmacy and Molecular Biotechnology, University of Heidelberg, Heidelberg, Germany

**Keywords:** *Caenorhabditis elegans*, aging, sulforaphane, DAF-16, insulin

## Abstract

The broccoli-derived isothiocyanate sulforaphane inhibits inflammation, oxidative stress and cancer, but its effect on healthspan and longevity are unclear. We used the *C. elegans* nematode model and fed the wildtype and 9 mutant strains ±sulforaphane. The lifespan, phenotype, pharyngeal pumping, mobility, lipofuscin accumulation, and RNA and protein expression of the nematodes were assessed by using Kaplan-Meier survival analysis, *in vivo* live imaging, fluorescence microscopy, and qRT-PCR. Sulforaphane increased the lifespan and promoted a health-related phenotype by increasing mobility, appetite and food intake and reducing lipofuscin accumulation. Mechanistically, sulforaphane inhibited DAF-2-mediated insulin/insulin-like growth factor signaling and its downstream targets AGE-1, AKT-1/AKT-2. This was associated with increased nuclear translocation of the FOXO transcription factor homolog DAF*-*16. In turn, the target genes *sod-3*, *mtl-1* and *gst-4*, known to enhance stress resistance and lifespan, were upregulated. These results indicate that sulforaphane prolongs the lifespan and healthspan of *C. elegans* through insulin/IGF-1 signaling. Our results provide the basis for a nutritional sulforaphane-enriched strategy for the promotion of healthy aging and disease prevention.

## INTRODUCTION

The risk of cancer, cardiovascular disease, and neurodegeneration rises dramatically later in life [1]. Therefore, the aging population is a global challenge for worldwide health systems [2]. During the aging process, systemic muscle cells gradually lose vitality, which causes declines in mobility, appetite and stress resistance [3]. Some longevity-related signaling pathways have been associated with aging-related diseases, such as insulin receptor and insulin-like growth factor (IGF) signaling, mitochondrial dysfunction, and dietary restriction [4]. To decode the underlying mechanisms of aging to define new options for the treatment of age-associated diseases, one focus is on healthy nutrition, which is believed to delay biological aging and typical diseases of the elderly. Several studies have described the isolation of natural substances from food plants and characterized them as suitable anti-aging agents; such substances include the phenol resveratrol from grapes and berries, the phenol curcumin from turmeric, the alkaloid berberine found in plants used in traditional Chinese medicine (TCM), the polyphenol chlorogenic acid from coffee and tea, and chlorophyll from green vegetables, among others [5–7].

**Figure 1:**
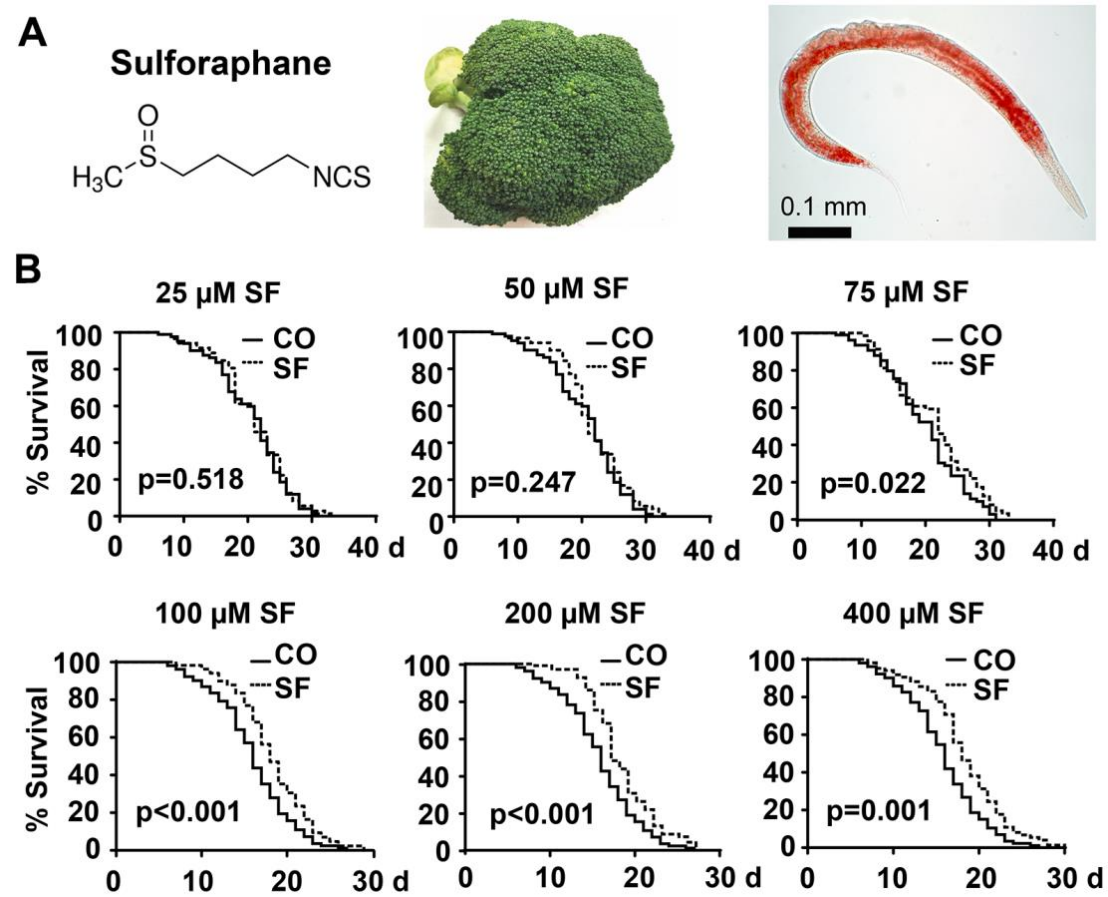
**Sulforaphane extends the lifespan.** (**A**) Schematic representation of the chemical structure of the isothiocyanate sulforaphane, which is enriched in broccoli, and an image of the nematode *C. elegans* - the scale bar indicates 0.1 mm. (**B**) Approximately 100 synchronized N2 wild-type *C. elegans* L4 larvae per group were transferred to fresh NGM plates and maintained at 20° C. This time point was considered day 0 (0 d) of the presented Kaplan-Meier curves. The plates contained 25 μM, 50 μM, 75 μM, 100 μM, 200 μM, or 400 μM sulforaphane (SF) or no sulforaphane (CO), as indicated. The worms were transferred daily from day 0 to 10 and every second day thereafter. The endpoint was defined as the day when all worms were dead. The numbers of living and censored worms are shown in Kaplan-Meier diagrams, and statistical significance was evaluated by the log-rank test. P=0.05 or below was considered statistically significant.

Epidemiological studies indicate that the regular consumption of broccoli and other vegetables of the Brassicaceae family, such as Brussels sprouts, cabbage, cauliflower, kale, swede and turnip, is associated with a reduced incidence of cancer [8], including pancreatic cancer [9–13]. The organosulfur isothiocyanate sulforaphane, found in *Brassicaceae* vegetables and especially in broccoli (Figure 1A), is one of the best-studied bioactive, anti-inflammatory agents and has been suggested to induce detoxifying enzyme production, cell cycle arrest, apoptosis and epigenetic regulation [14, 15]. Additionally, sulforaphane has been shown to reduce hepatic glucose production and to improve glucose control in patients with type 2 diabetes [16]. This is interesting because diabetes is discussed as a risk factor for pancreatic cancer [17]. Excitingly, the lifespan of the red flour beetle *Tribolium castaneum* was found to increase by approximately 30% when 1% lyophilized broccoli was fed together with the regular diet, not only under physiological conditions but also under heat stress conditions [18]. More specifically, crude extracts of *Brassica chinensis*, also known as pak choy, which is one of the most widely consumed *Brassica* vegetables in Asian countries, have been reported to enhance antioxidant activity in a cell-free system and exert anti-aging effects in the nematode *Caenorhabditis (C.) elegans* [19].

*C. elegans* is one of the most widely used models for aging research due to its short lifespan of approximately 4 weeks and highly conserved key aging-related signaling molecules [20]. In *C. elegans,* DAF-2 is the highly conserved nutrient-sensing insulin/IGF receptor, and its major downstream transcription factor DAF-16, a homologue of human FOXO [21], regulates the expression of a variety of genes, such as *sod-3* (superoxide dismutase 3), *gst-4* (glutathione S-transferase 4), *dod-3* (downstream of DAF-16), *ctl* (catalase-2) and *mtl-1* (metallothionein-1) [22]. These DAF-16 target genes are involved in the regulation of *C. elegans* lifespan [23] by influencing longevity, stress resistance, development, and the resting dauer diapause [24, 25]. This is in line with the finding of a benefit of chlorogenic acid, or chlorophyll, that regulates DAF-16/FOXO-dependent insulin-like signaling, promotes antioxidant resistance and thereby improves the health and longevity of *C. elegans* [6, 7].

Here, we asked whether sulforaphane may influence the lifespan and healthspan of *C. elegans*. We found that sulforaphane significantly extends the lifespan of *C. elegans* and delays age-related phenotype changes. The analysis of wild-type *C. elegans* and 9 mutant strains revealed that sulforaphane inhibited DAF-2 insulin/insulin receptor signaling and thereby increased DAF-16 nuclear translocation, resulting in the expression of the *sod-3*, *mtl-1* and *gst-4* target genes, which are known mediators of longevity in *C. elegans*. These results indicate that the consumption of broccoli or supplements obtained from this vegetable may be of high interest in anti-aging research and cancer prevention.

## RESULTS

### Sulforaphane prolongs longevity

**Figure 2:**
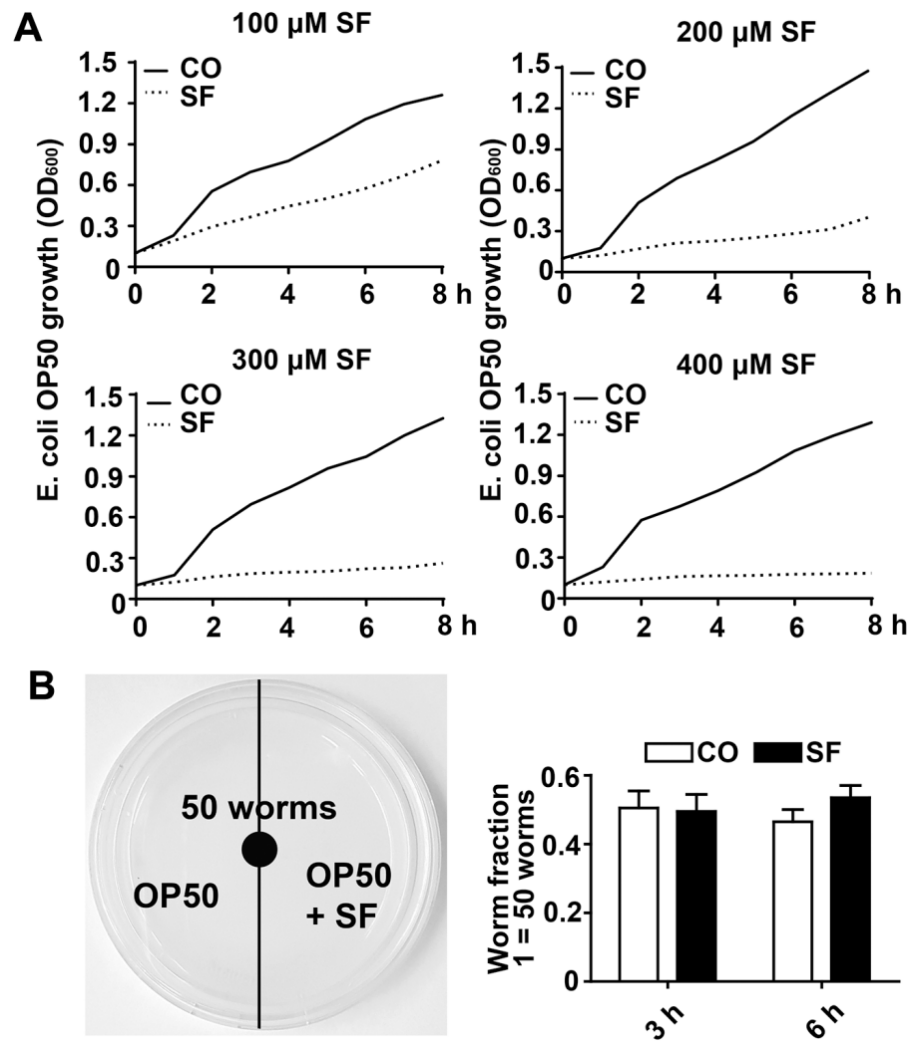
**Low-dose sulforaphane does not inhibit OP50 bacterial growth and *C. elegans* food preferences.** (**A**) A single colony of *E. coli* OP50 was allowed to grow in 3 ml of LB broth medium in a 37° C shaker overnight. The overnight culture was diluted to an OD_600_ of approximately 0.1. Then, 15 ml aliquots of this dilution were pipetted into 50 ml falcon tubes in the presence or absence of 100 μM, 200 μM, 300 μM, or 400 μM sulforaphane (SF) as indicated, followed by incubation in a 37° C shaker. The OD_600_ was measured every 30 min up to 8 h, and the resulting growth curves are presented as *E. coli* OP50 growth (OD_600_). (**B**) Fifty wild-type L4 larvae were placed in the center of an NGM plate with *E. coli* OP50 bacteria, where the bacteria on the left side contained 100 μM sulforaphane (SF), whereas those on the right side did not (CO), as indicated in the scheme on the left. The number of *C. elegans* worms on each side of the agar plate was determined by picking and counting the nematodes 3 h and 6 h later. The worm fraction was calculated as the ratio of the number of worms crawling on each side of the plate. The initial number of 50 worms was set as 1. The data are presented in the diagram on the right. Mean values ±SD are given.

We wondered whether sulforaphane might affect aging processes and performed Kaplan-Meier survival studies by feeding wild-type 3-day-old *C. elegans* larvae (L4) with *Escherichia coli (E. coli)* OP50 food bacteria, either alone or in the presence of 25 μM, 50 μM, 75 μM, 100 μM, 200 μM, or 400 μM sulforaphane daily until the worms had died. The number of living worms was determined daily until no living worms could be found. Whereas the wild-type adult worms lived 16 days on average, the survival of the worms was significantly increased by 18.2%, 13.31% and 15.46% by exposure to the 100 μM, 200 μM and 400 μM concentrations, respectively, whereas lower sulforaphane concentrations had no effect on survival (Figure 1B, Tables 1, 2). Since 100 μM sulforaphane was the lowest effective concentration and fits to the oral administered sulforaphane concentration of 10 mg/kg (56.47 μM/L), which has been used in other animal models [26, 27], we chose this concentration for all subsequent experiments. To rule out the possibility that sulforaphane might have inhibited the growth of OP50 bacteria and might thereby have caused dietary restriction-induced longevity, we determined the minimum inhibitory concentration (MIC), at which sulforaphane inhibits OP50 growth. OP50 bacteria were treated with sulforaphane in the range of 8 μM to 1000 μM in 96-well plates using the double dilution method. On the basis of the visual inspection of the turbidity of the OP50 medium, we defined a concentration of 700 μM sulforaphane as the MIC (data not shown). In parallel, we generated growth curves via the treatment of OP50 bacteria with different sulforaphane concentrations in the range of 100 μM to 400 μM and found no effect on bacterial growth (Figure 2A). In a second control experiment, we wanted to exclude the possibility that *C. elegans* prefers sulforaphane-treated OP50 bacteria as food. Therefore, we performed an avoidance assay. While one side of an agar plate contained OP50 bacteria plus sulforaphane, the other side contained only OP50 bacteria. The *C. elegans* worms were placed exactly in the middle of the plate. After 3 or 6 h, the worms that had migrated to the left and right side of the agar plate were picked and counted (Figure 2B). No preference in migration to either side was found, suggesting that *C. elegans* does not avoid or prefer sulforaphane-treated OP50 food bacteria. These results indicate that sulforaphane might have affected the longevity of *C. elegans* directly.

**Table 1:** Characterization of C. elegans strains used in the present study.

**Strain name and WB ID**	**Genotype**	**Description**	*Mean life span (days)		**Ref**
			**CO**	**SF**	
N2 00000001	*wildtype*	Isolated 1951 from mushroom compost in an urban garden by W. L. Nicholas in Bristol. Development time: ~3 days. Brood size: ~330 eggs. Lifespan: ~3 weeks.	16.023	18.702	[52]
CB1370 *00004309*	*daf-2(e1370) III.*	DAF-2/Insulin/IGF-1 receptor mutant. Made by Edgar B by EMS mutagen. Slower aging and longevity; temperature sensitive dauer constitutive, maintains at 15° C.	35.386	35.340	[30]
DA1116 00005548	*eat-2(ad1116)II.*	Nicotinic acetylcholine receptor mutant. Made by D. Raizen by EMS mutagen. Slower eating+pumping, long-lived; enhanced embryonic lethality.	22.609	27.292	[31, 32]
VC199 00035578	*sir-2.1(ok343)IV.*	Deacetylase sirtuin mutant. Superficially wild type. Made by B. J. Allan by UV/TMP mutagen.	18.416	19.532	[33]
MQ887 00026670	*isp-1(qm150)IV.*	Mitochondrial Complex III mutant. Slow development and behavior. Long lived.	23.533	30.223	[34, 35]
CF1038 00004851	*daf-16(mu86)I.*	FOXO/DAF-16 mutant. Made by C. Kenyon lab by UV/TMP mutagen. Dauer defective and short lived.	14.323	14.798	[36]
GR1309 *00007897*	*daf16(mgDf47)I;* *daf-2(e1370) III.*	Double mutant of DAF-16 (mgDf47) and DAF-2 (e1370). A mgDf47 deletion ~8 kb of the daf-16 gene; Made by G. Ruvkun by γ-irradiation. The e1370 allele substitution was made by the Jonathan Hodgkin lab. Dauer defective and short lived.	14.931	14.352	[36]
RB712 00031447	*daf-18(ok480) IV.*	PTEN mutant. Homozygous worm. Made by the A. Rougvie lab by UV/TMP mutagen. Superficially wild type and long lived.	12.874	12.763	[53] [54]
TJ105200034902	*age-1(hx546) II.*	PIK3C mutant. Made by the A. Rougvie lab by UV/TMP mutagen. Superficially wild type, stress tolerant and long lived.	25.597	25.034	[58]
RB75900031473	*akt-1(ok525)V.*	AKT mutant. Homozygous worms. Made by the A. Rougvie lab by UV/TMP mutagen. Superficially wild type and long lived.	26.506	26.082	[55]
VC20400035581	*akt-2(ok393)X.*	AKT mutant. Made by the A. Rougvie lab by UV/TMP mutagen. Superficially wild type and long lived.	23.392	23.925	[55] [56]
TJ35600005218	*pGP30(daf-16p, daf-116a/b, GFP*	The daf-16 gene is fused to a GFP reporter and driven by the daf-16 promoter. Made by the A. Rougvie lab by γ-irradiation. Superficially wild type.			[37, 38]
CL1553 00004861	*(pAD76) sod-3p::GFP*	The sod-3 gene is fused to a GFP reporter and driven by the sod-3 promoter. SOD-3 is expressed in several structures, including head; anterior intestine; pharynx; tail; and vulva.			[59]
CL2166 00005102	*(pAF15)gst-4p::GFP::NLS*	The gst-4 gene is fused to a GFP reporter and driven by the gst-4 promoter. Is expressed in body wall musculature; hypodermis; muscle cell; and pharynx.			[59]

*Mean life span: Corresponds to the mean life span as measured in the present study.

**Table 2:** Sulforaphane effects on lifespan of *C. elegans* strains.

**C. elegans strain**	**Tem-per-ature**	**Fig. No.**	**Exp.No.**	**Sulforaphane (μM)**	**Mean lifespan ±SEM (days)**	**% Change mean lifespan**	**P value vs CO**
**N2 wildtype**	20° C	1B	1	0	16.02±0.49	16.72	<0.001
				100	18.70±0.54		
			2	0	15.94±0.47	19.09	0.002
				100	18.98±0.63		
			3	0	16.19±0.52	14.37	<0.001
				100	18.51±0.54		
		1B	1	0	15.82±0.47	15.30	<0.001
				200	18.24±0.23		
			2	0	15.72±0.49	10.84	0.039
				200	17.42±0.57		
			3	0	15.96±0.50	11.75	0.022
				200	17.83±0.58		
		1B	1	0	15.75±0.47	16.01	0.001
				400	18.27±0.53		
			2	0	14.11±0.42	14.91	0.002
				400	16.21±0.44		
			3	0	12.77±0.48	27.30	<0.001
				400	16.25±0.74		
**CB1370*daf-2(e1370)III***	15° C	5B	1	0	35.39±0.71	-	0.275
				100	35.34±0.64		
			2	0	35.90±1.03	-	0.849
				100	37.43±0.87		
			3	0	35.94±0.96	(7.75) -	0.003
				100	33.15±0.89		
**DA1116*eat-2(ad1116)II***	20° C	5B	1	0	22.61±0.64	20.71	<0.001
				100	27.29±0.96		
			2	0	24.30±0.91	11.28	0.030
				100	27.05±0.94		
			3	0	24.34±0.90	10.86	0.016
				100	26.99±1.01		
**VC199*sir-2.1(ok343)IV***	20° C	5B	1	0	18.42±0.21	6.06	0.003
				100	19.53±0.35		
			2	0	17.84±0.36	4.82	0.056
				100	18.70±0.43		
			3	0	19.17±0.44	6.28	0.043
				100	20.38±0.47		
**MQ887isp-1(qm150)*IV***	20° C	5B	1	0	23.53±0.89	28.32	<0.001
				100	30.23±0.98		
			2	0	24.64±1.22	23.07	<0.001
				100	30.36±1.37		
			3	0	24.16±1.28	25.62	0.001
				100	30.36±1.38		
**CF1038*daf-16(mu86)I***	20° C	6A	1	0	14.32±0.23	(3.35) -	0.054
				100	14.80±0.24		
			2	0	14.63±0.36	-	0.055
				100	15.50±0.42		
			3	0	13.87±0.31	-	0.429
				100	14.11±0.34		
**GR1309*daf-16(mgDf47)I;daf-2(e1370) III***	20° C	7A	1	0	14.93±0.38	-	0.244
				100	14.35±0.34		
			2	0	14.83±0.57	-	0.423
				100	14.33±0.50		
			3	0	14.46±0.48	-	0.667
				100	14.37±0.48		
**RB712, *daf-18(ok480) IV***	20° C	7A	1	0	12.87±0.31	-	0.674
				100	12.76±0.25		
			2	0	13.01±0.36	-	0.816
				100	13.20±0.31		
			3	0	12.94±0.24	-	0.514
				100	12.98±0.20		
**TJ1052*age-1(hx546) II***	20° C	7A	1	0	25.58±0.68	-	0.631
				100	25.03±0.62		
			2	0	24.69±0.74	-	0.642
				100	25.77±0.61		
			3	0	25.10±0.51	-	0.943
				100	25.50±0.43		
**RB759*akt-1(ok525)V***	20° C	7A	1	0	26.51±0.51	-	0.430
				100	26.08±0.47		
			2	0	24.01±0.71	-	0.230
				100	25.36±0.71		
			3	0	28.92±0.62	-	0.078
				100	27.09±0.62		
**VC204*akt-2(ok393)X***	20° C	7A	1	0	23.39±0.28	(2.31) -	0.051
				100	23.93±0.31		
			2	0	23.37±0.39	-	0.082
				100	24.00±0.43		
			3	0	23.42±0.42	-	0.184
				100	24.11±0.52		

Summary of mean lifespan and statistical analysis (p-values) for lifespan experiments including all different treatments displayed in Figures 1, 5, 6 and 7. P-values less than 0.05 were considered statistically significant, demonstrating that the lifespan of the populations is different. The mean lifespan values were calculated by a log-rank (Kaplan-Meier) statistical test. p-values were calculated for individual experiments, each consisting of control and treatment groups. The total number of worms in each individual experiment was about 100. All statistical evaluations were calculated by using SPSS.

(-) not calculated because the standard deviation was >0.05.

### Sulforaphane increases healthspan

**Figure 3:**
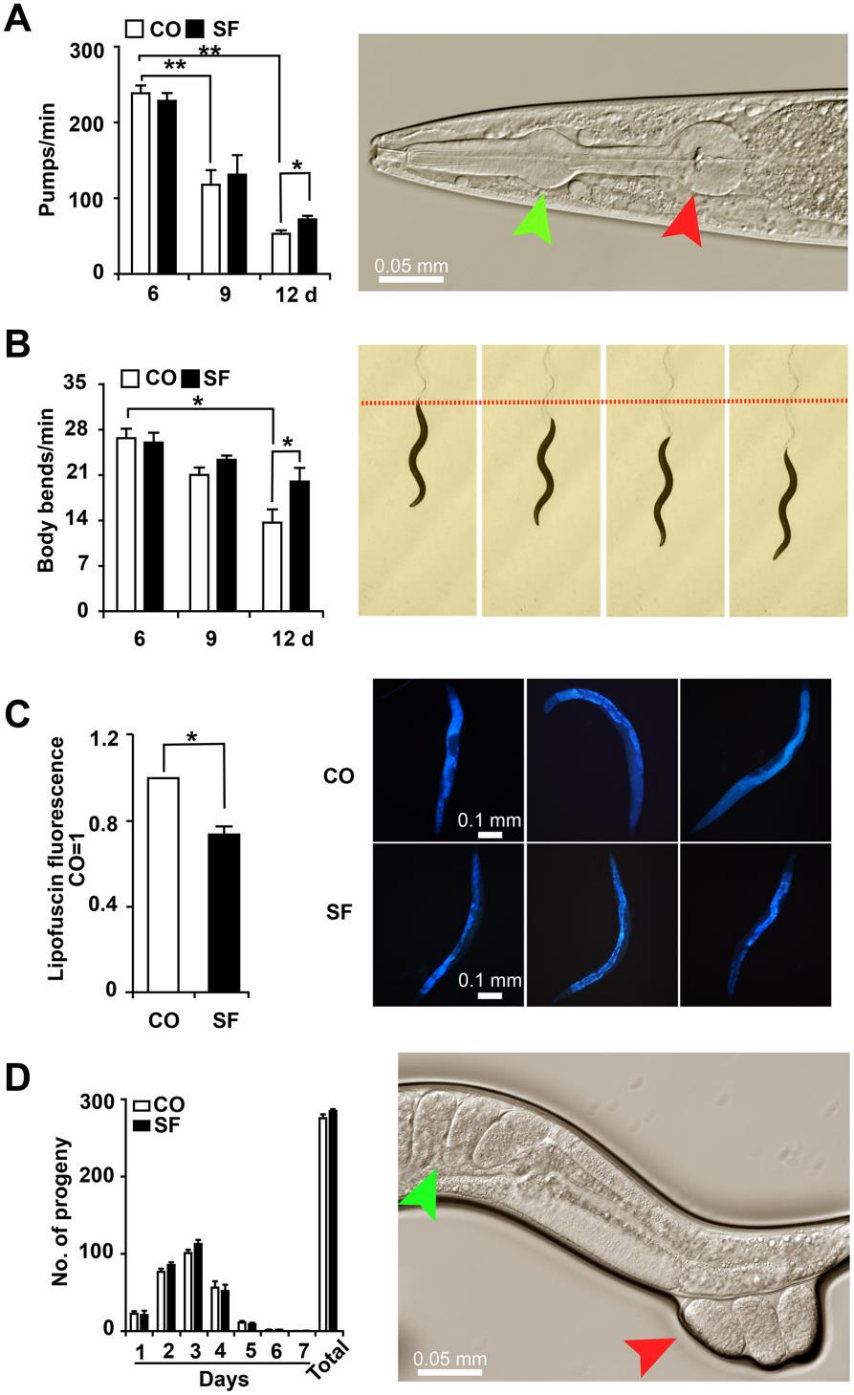
**Sulforaphane delays age-associated physiological decline.** (**A**) L4 larvae were exposed to 100 μM sulforaphane (SF) or not (CO), and 20 worms per group were used for evaluation. Pharyngeal pumping was measured on days 6, 9 and 12 by the evaluation of the opening of the corpus (anterior pharynx, green arrow, image on the right) and the terminal bulb (red arrow), which contract and relax synchronously during pharyngeal pumping. The pump frequency per minute was calculated and is shown in the diagram on the left. (**B**) In L4 larvae treated as described above, the number of body bends (images on the right) per minute was evaluated (diagram on the left) to assess mobility. (**C**) At day 12 after sulforaphane treatment, blue autofluorescence, representing lipofuscin accumulation (images on the right), was detected by fluorescence microscopy. The blue autofluorescence in the figure indicates the accumulation of lipofuscin. The relative fluorescence intensity was evaluated by using ImageJ software, and the fluorescence of the untreated nematodes was set as 1. The scale bar indicates 0.1 mm. (**D**) L4 larvae were treated with sulforaphane (SF) or not (CO). Twenty-four hours later (Day 1), the number of eggs laid was counted under the microscope daily for 7 days. The number of eggs laid each day and the total number of eggs are shown in the diagram on the left. The green arrow in the diagram on the right indicates eggs in a worm, and the red arrow indicates eggs that are being laid by the worm. The data are shown as the mean ± SD as evaluated by Student's t test using Prism 6.0. ^*^P< 0.05, ^**^P < 0.01.

To assess whether sulforaphane affects not only lifespan but also health-span, we analyzed age-associated physiological functions. For the evaluation of eating and appetite, *C. elegans* L4 larvae were treated with sulforaphane or were left untreated in the control group. The pumping frequency of the terminal pharyngeal bulb of each worm was documented by microscopy and video recordings at days 6, 9 and 12 after treatment. Young 6-day-old control nematodes exhibited a high mean rate of 239 pumps per minute, which was significantly reduced to 118 pumps at day 9 and to 53 pumps at day 12 (Figure 3A). Whereas sulforaphane cotreatment did not significantly alter the frequency of pharyngeal pumping in young worms at days 6 and 9, it significantly increased it to 72 pumps per minute at day 12. Likewise, the mobility represented by the number of body bends per minute was evaluated by microscopy. The number of body bends per minute became continuously and significantly smaller with increasing age, as measured from day 6 to 12 (Figure 3B). Whereas sulforaphane had no significant effect on the frequency of body bending at days 6 and 9, a significant increase from 14 to 20 body bends per minute was observable at day 12. This corresponds to a 43% higher body bending rate in older (12-day-old) worms. Next, we evaluated the effect of sulforaphane on the accumulation of lipofuscin, which is considered an aging (or “wear-and-tear”) pigment, as a hallmark of aging. The autofluorescence of intestinal lipofuscin in 15-day-old *C. elegans* worms was detected by the application of blue excitation light (405-488 nm) and fluorescence microscopy. Compared to the autofluorescence of control worms, which was set as 1, sulforaphane significantly decreased autofluorescence to 0.74, which corresponds to a reduction of 26.4% (Figure 3C). Moreover, the number of progeny was determined by a brood size assay. Synchronized L4 larvae were isolated and incubated in the presence or absence of sulforaphane for 24 h. Then, the number of eggs laid each day was counted for 7 days. The number of eggs laid was highest at days 2 and 3 and then continuously decreased to close to 0 at day 7, as expected (Figure 3D). The brood sizes of the control group and sulforaphane-treated group were 275.7 and 284.8, respectively, which did not represent a significant difference.

### Sulforaphane enhances stress resistance

**Figure 4:**
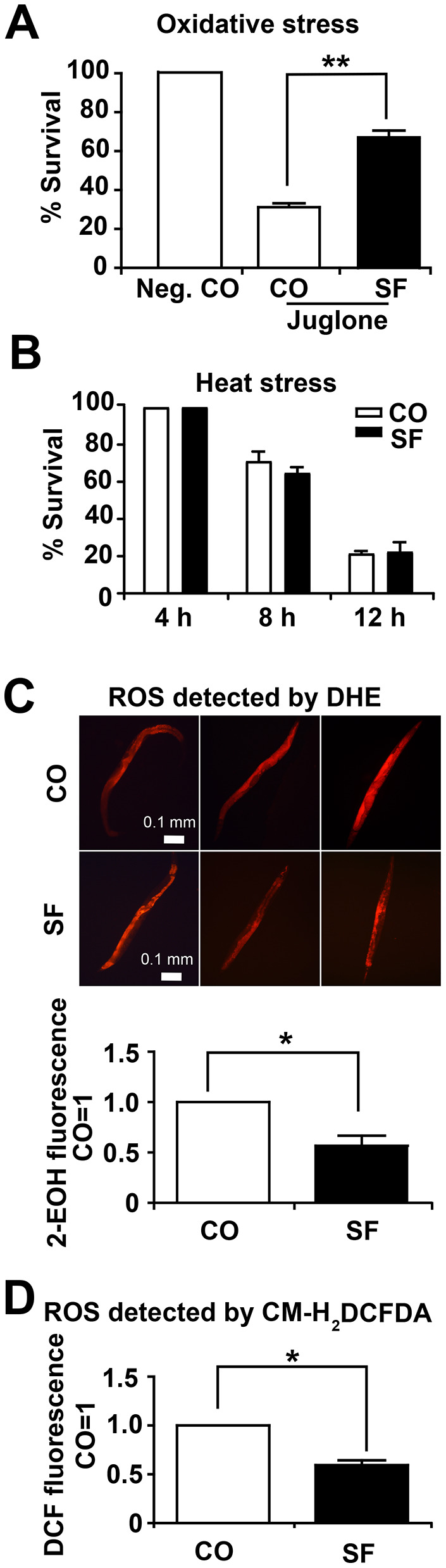
**Sulforaphane increases stress resistance.** (A) Approximately 100 N2 wild-type *C. elegans* L4 larvae per group were transferred to liquid NGM medium containing 120 μM juglone (Jug), either alone together with 100 μM sulforaphane (SF), whereas the negative control (Neg. CO) did not contain either. The worms were maintained at 20° C for 24 h, followed by counting the number of surviving worms, which was set as 100 in the negative control, and the percentage of surviving control and sulforaphane worms was calculated related to the negative control (% survival). (B) Approximately 100 wild-type *C. elegans* L4 larvae were transferred to NGM agar medium containing 100 μM sulforaphane (SF) or not (CO), followed by incubation at 35° C for 4 h, 8 h and 12 h, as indicated. Thereafter, the number of surviving worms was evaluated and is presented as the % survival. (C) Approximately 100 L4 *C. elegans* N2 wild-type larvae were grown in 100 μM sulforaphane (SF) or were left untreated (CO). Ten days later, 20 adult worms were selected, washed and incubated in 5 μM DHE in M9 buffer in the dark at 20° C for 1 h. After washing, the worms were mounted on a glass slide, paralyzed with a drop of 10 mM sodium azide, and the total fluorescence of each worm was analyzed by fluorescence microscopy, where that the intensity of fluorescence was dependent on intracellular oxidation of DHE by superoxide/ROS to form 2-hydroxyethidium (2-EOH). Representative images at 100× magnification are shown, and the scale bar indicates 0.1 mm. The fluorescence intensity was analyzed with ImageJ, and the fluorescence of the control was set as 1. (D) Fifty adult *C. elegans* N2 wild-type worms were treated and selected as described above. After washing, proteins were harvested, and 50 μl of protein lysate per group was incubated with a 20 μM concentration of the fluorescent dye CM-H_2_DCFDA for 30 min at 20° C in the dark. CM-H_2_CFDA is oxidized by superoxide/ROS into the fluorescent dye DCF, which was quantified through the use of a microplate reader with excitation at 485 nm and emission at 520 nm. The data are expressed as the mean ± SD. *P <0.05, **P<0.01.

To test the effects of sulforaphane on the viability of *C. elegans* under oxidative stress and heat stress, L4 larvae were treated with 100 μM sulforaphane for 2 days or were left untreated in the control. Then, the nematodes were exposed to oxidative stress by transfer to liquid medium containing a 120 μM concentration of the pro-oxidant juglone. Twenty-four hours later, the number of surviving worms was evaluated by picking and counting live worms. Compared to the survival of untreated control worms, which was set as 100%, juglone reduced survival to 31%, whereas 72% of sulforaphane and juglone-cotreated worms remained alive, which was a significant difference (Figure 4A). For the evaluation of heat stress, nematodes were pretreated with sulforaphane as described above and then incubated at 35° C for 4 h, 8 h and 12 h. The evaluation of the surviving fraction revealed that 100%, 71% and 21% of the control worms remained alive after 4 h, 8 h and 12 h, respectively. However, sulforaphane did not increase heat stress resistance under these conditions (Figure 4B). For the evaluation of reactive oxygen species (ROS) levels, 3-day-old L4 larvae were treated with sulforaphane for 10 days, followed by the incubation of the live worms in fluorescent dihydroethidium (DHE) for 1 h. DHE is taken up by the worms and oxidized by superoxide to form 2-hydroxyethidium (2-EOH). Thereafter, we examined the paralyzed worms with red excitation light (530-620 nm) under fluorescence microscopy as described previously [28]. According to setting the fluorescence of the control worms as 1, we found a significant reduction to 0.57 in the sulforaphane-treated worms, which corresponds to a reduction of 43% (Figure 4C). Likewise, we detected ROS by the incubation of protein lysates with the fluorescence probe CM-H_2_DCFDA, which passively diffuses into cells, where its acetate groups are cleaved by intracellular esterases and its thiol-reactive chloromethyl group reacts with intracellular glutathione and other thiols [29]. Subsequent oxidation yields a fluorescent adduct, which we quantified through the use of a microplate reader with excitation at 485 nm and an emission at 520 nm. By setting the fluorescence intensity of untreated *C. elegans* worms to 1, we found a significant reduction to 0.6 in sulforaphane-treated nematodes (Figure 4D), which corresponds to a difference of 41%.

### DAF-2/insulin/IGF-1 signaling is required for sulforaphane-induced longevity

**Figure 5:**
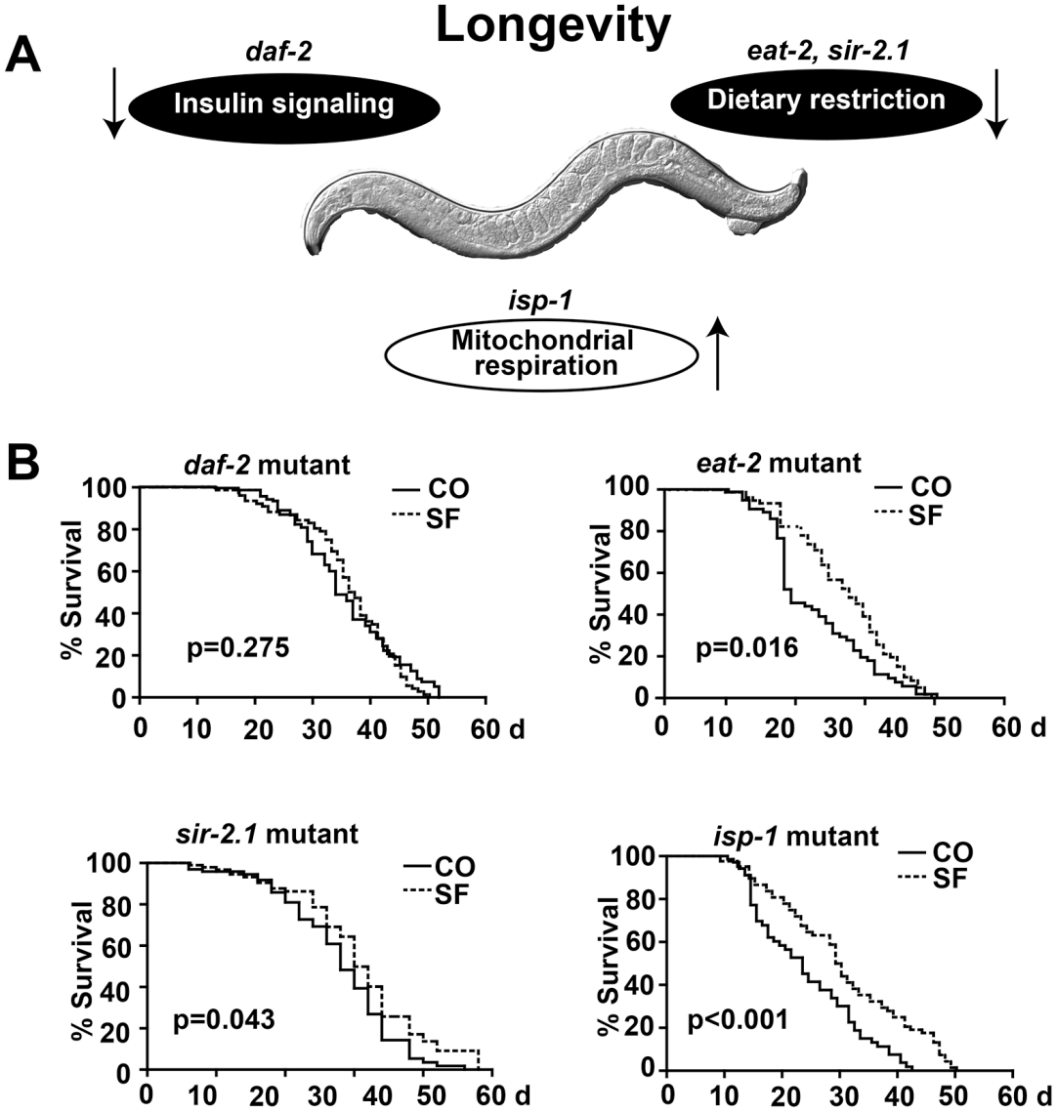
**DAF-2/insulin/IGF-1 signaling is required for sulforaphane-mediated longevity.** (**A**) Schematic representation of signaling cascades that are known to regulate longevity in *C. elegans*, together with the names of the respective mutant *C. elegans* strains are shown, e.g., reduced DAF-2/insulin signaling, reduced eat-2/sir-2.1 dietary restriction signaling, and enhanced isp-1 mitochondrial respiration. (**B**) Kaplan-Meier survival curves were generated using *C. elegans* strains with mutations in *daf-2*, *eat-2*, *sir-2.1* and *isp-1*. The worms were fed with OP50 bacterial only (CO) or with 100 μM sulforaphane plus OP50 bacteria (SF), as described in Fig. 1. The *daf-2* mutant *daf-2(e1370) III* strain was cultivated at 15° C, and all other strains were cultivated at 20° C. *daf-2(e1370) III: P>*0.05, *eat-2(ad1113) II:* P< 0.001, *sir-2.1(ok434) IV:* P< 0.01, *isp-1(qm150) IV*(D): P< 0.001.

To elucidate the underlying pathway of sulforaphane-induced longevity, we focused on signaling cascades that were recently associated with the longevity of *C. elegans*, specifically the inhibition of DAF-2/insulin/IGF-1 receptor signaling (*daf-2* mutant), dietary restriction (*eat-2*, *sir-2.1* mutants), or increased mitochondrial respiration (*isp-1* mutant) (Figure 5A, Table 1). The treatment of the long-lived *C. elegans* DAF-2 mutant [30] with sulforaphane did not significantly alter its mean lifespan of 35.4 days (Figure 5B, Tables 1, 2). In contrast, the sulforaphane treatment of the long-lived nicotinic acetylcholine receptor mutant eat-2, which mimics dietary restriction by slowing eating and pharyngeal pumping [31, 32], further enhanced the mean lifespan significantly from 22.6 days to 27.3 days. The sulforaphane treatment of the deacetylase sirtuin mutant sir-2, which exhibits altered NAD^+^-dependent protein deacetylases that are responsive to metabolic changes, including nutrient/energy availability and cellular stress [33], significantly increased the mean lifespan from 18.4 days to 19.5 days. Finally, the sulforaphane treatment of the long-lived mitochondrial complex III mutant isp-1 which displays slow development and behavior [34, 35], significantly increased the mean lifespan of 24 days to 30.2 days. Since sulforaphane was able to prolong survival in all mutants except the DAF-2 mutant, this speaks to the involvement of DAF-2/insulin/IGF-1 receptor signaling in the life-prolonging function of sulforaphane.

### DAF-2/insulin/IGF-1 receptor signaling is required for sulforaphane-induced stress resistance

To evaluate whether DAF-2/insulin/IGF-1 signaling is required for the sulforaphane-mediated stress resistance, *daf-2* mutant (CB1370) L4 larvae were treated with 100 μM sulforaphane for 2 days or were left untreated in the control. Then, the CB1370 worms were cultured in liquid medium containing 120 μM of the bioactive pro-oxidant juglone for induction of oxidative stress. After 24 hours, the number of surviving worms was evaluated by counting live worms. Compared with the survival rate of untreated control worms (set as a 100% negative control), juglone reduced the survival rate to 84%, while the survival rate of sulforaphane and juglone-treated worms was 81%, with no significant differences between groups (Supplementary Figure 1A). To assess heat stress, daf-2 mutant worms were pretreated with sulforaphane as described above, and then incubated at 35° C for 4 h, 8 h, and 12 h. The evaluation of the surviving worms indicated that 100%, 87% and 68% of the control worms were alive after 4 h, 8 h and 12 h, respectively. The survival rates of the sulforaphane group were comparable and suggest that sulforaphane did not increase heat stress resistance under these conditions (Supplementary Figure 1B). However, whereas 12 h after heat stress 80% of daf-2 mutant *C. elegans* nematodes were alive, only 20% of wildtype worms survived (compare Figure 4B and Supplementary Figure 1B). These data suggest that daf-2 mutant worms are more resistant to heat stress. To assess ROS accumulation, L4 larvae of daf-2 mutants were left untreated or were treated with sulforaphane for 10 days. Then the live worms were selected and incubated with the fluorescent dihydroethidine (DHE) for 1 h, followed by fluorescence microscopy with red excitation light of 530-620 nm. By setting the fluorescence intensity of control worms to 1, sulforaphane slightly increased the fluorescence intensity, although this difference was not significant (Supplementary Figure 1C).

### DAF-16/FOXO mediates sulforaphane-induced longevity and stress resistance

**Figure 6:**
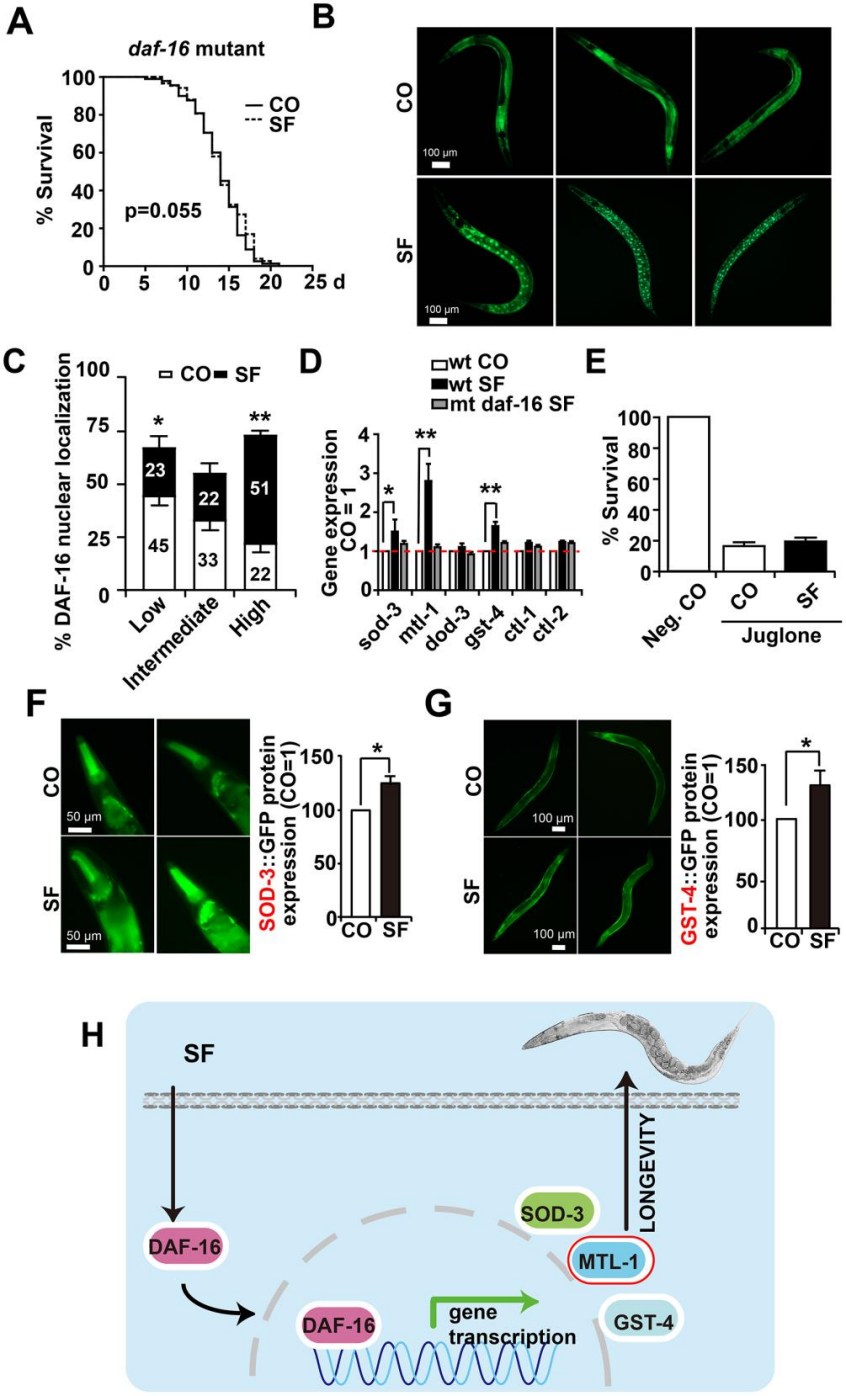
**DAF-16 mediates sulforaphane-induced longevity and stress resistance.** (**A**) Approximately 100 L4 larvae from the *C. elegans* CF1038 *daf-16(mu86)I strain*, carrying a mutant *daf-16* gene (*daf-16* mutant), were fed in the presence or absence (CO) of 100 μM sulforaphane (SF), and a Kaplan-Meier survival curve was generated as described in Figure. 1 and Materials and Methods. P=0.055, according to a log-rank test. (**B**) Adult TJ356 worms with a daf-16-GFP fusion gene were treated with sulforaphane for 3 days (SF) or were left untreated (CO), followed by the detection of green fluorescence in 20 worms per group by fluorescence microscopy. The bright green fluorescent dots indicate the nuclear localization of DAF-16. The scale bar indicates 100 μm. (**C**) The number of spots was counted and is represented in the diagram. The following scoring of the fluorescence intensity was applied: low (number of green fluorescent spots <50%), intermediate (number of green fluorescent spots ≈50%), and high (number of green fluorescent spots >50%). (**D**) Approximately 200 synchronized *C. elegans* L4 wild-type larvae received a regular diet (wt CO) or were fed in the presence of sulforaphane (wt SF). Additionally, L4 larvae of the *daf-16(mu86)I* strain, carrying a daf-16 mutation (*mt daf-16*), were fed in the presence of sulforaphane (mt daf-16 SF). Three days later, living worms were selected, and total RNA was extracted. A qRT-PCR assay with specific *C. elegans* primers (Table 3) was performed to detect the expression of the DAF-16-target genes *sod-3*, *mtl-1*, *dod-3*, *gst-4*, *ctl-1*, and *ctl-2*. The expression levels in untreated control worms were set as 1. (**E**) Approximately 100 synchronized L4 larvae of the *daf-16(mu86)I C. elegans* strain were fed OP50 bacteria in the absence (Neg. CO) or presence of sulforaphane (SF), in the presence or absence of a 120 μM concentration of the oxidative chemical juglone, as indicated. Fourteen days later, the surviving worms of each group were selected and counted. (**F**) L4 CF1553 worms with a SOD-3::GFP fusion gene were fed with sulforaphane for 3 days (SF) or were left untreated (CO), followed by the detection of green fluorescence in 20 worms per group by fluorescence microscopy, indicating a high expression of SOD-3 in the head and foregut of *C. elegans*. The fluorescence intensity was analyzed by imageJ, and the fluorescence of the control was set to 1. Representative images at 100× magnification are shown on the left, and the scale bar indicates 50 μm. (**G**) L4 CL2166 worms with a GST-4::GFP fusion gene were treated and analyzed as described above. Representative images at 100× magnification are shown on the left, and the scale bar indicates 100 μm. *P<0.05. (**H**) Cartoon to demonstrate that sulforaphane mediates the nuclear translocation of the DAF-16 transcription factor, where it binds to the promoter regions of its target genes and thereby activates SOD-3, MTL-1 and GST-4, which mediate the stress resistance and longevity of *C. elegans.* **P<0.01; *P<0.05.

To further elucidate our findings, we examined whether sulforaphane might target the DAF-16/FOXO transcription factor downstream of DAF-2. We used the CF1038 *daf-16(mu86)I. C. elegans* strain, harboring a mutant form of daf-16 [36], and evaluated survival in the presence or absence of sulforaphane. However, sulforaphane did not significantly alter the mean lifespan of 14.32 days observed in untreated worms (Figure 6A, Tables 1, 2). These data suggest that the transcriptional activity of DAF-16 is required for sulforaphane-mediated longevity. To determine whether sulforaphane induces the nuclear translocation and, thus, the transcriptional activity of DAF-16, we used the worm strain TJ356 (DAF-16::GFP) [37, 38], which is characterized by the fusion of the *daf-16* gene to a GFP reporter driven by the daf-16 promoter. We started with *C. elegans* L4 larvae and fed them with OP50 bacteria in the presence or absence of sulforaphane. Three days later, the expression and cellular location of GFP were examined by fluorescence microscopy. Whereas GFP fluorescence could be detected throughout the cytosol in the worms, sulforaphane-treated worms exhibited green fluorescent spots indicating the nuclear translocation of DAF-16. For the analysis and scoring of the nuclear localization of DAF-16, the worms were divided into low-, intermediate-, and high-fluorescence groups. If the green fluorescent nuclei within a worm accounted for <50%, =50% or >50% of the total nuclei, the result was scored as low, intermediate, or high fluorescence, respectively. A significantly increased number of highly fluorescent nuclei was found an in sulforaphane-treated worms compared to control worms (Figure 6B, 6C). In a control experiment, we excluded interference from endogenous fluorescence by ensuring that the worms had consumed sufficient food prior to fluorescence analysis because endogenous fluorescence in this case mainly refers to green autofluorescence of the intestine, and food intake can reduce this strong intestinal autofluorescence. Finally, we examined the gene expression of *daf-16* target genes through the use of specific primer pairs for the *C. elegans* genes *sod-3*, *mtl-1*, *dod-3*, *gst-4*, *ctl-1* and *ctl-2* (Table 3). The expression levels of *sod-3*, *mtl-1* and *gst-4* significantly increased after the feeding of wild-type N2 *C. elegans* nematodes with sulforaphane as measured by qRT-PCR, while the expression levels of the other genes did not change significantly (Figure 6D). In contrast, sulforaphane feeding did not significantly alter the expression levels of any of these genes in CF1038 *C. elegans* worms carrying a mutant daf-16 gene, nor did it protect against juglone-induced oxidative stress (Figure 6E), as it was observed in wild-type N2 *C. elegans* nematodes (compare Figure 4A). Subsequently, the protein expression of the sod-3 and gst-4 genes was evaluated indirectly by the use of the GFP reporter strains CF1553 and CL2166, which harbor sod-3-GFP or gst-4-GFP fusion genes, respectively. The detection of GFP fluorescence by microscopy revealed that the protein expression of SOD-3 and GST-4 significantly increased after treatment with sulforaphane (Figure 6F, 6G), thus confirming the former mRNA expression data. These data suggest that sulforaphane induces the nuclear translocation of the FOXO/DAF-16 transcription factor, which in turn activates the promoters of *sod-3*, *mtl-1* and *gst-4* to induce the expression of these genes to mediate stress resistance and longevity (Scheme Figure 6H).

**Table 3:** Primer sequences for *C. elegans* candidate genes.

**Gene**	**Type**	**Sequence**
***sod-3***	F	5’- TCGCACTGCTTCAAAGCTTGTTCA A -3’
	R	5’- CCAAATCTGCATAGTCAGATGGGAGAT -3’
***ctl-1***	F	5’- TCGTTCATGCCAAGGGAGC-3’
	R	5’- GATCCCGATTCTCCAGCGAC-3’
***ctl-2***	F	5’- GAAGGTGTTGGATACCGGGG-3’
	R	5’- GGATGAGTGCCTTGACACGA-3’
***gst-4***	F	5’- CCCATTTTACAAGTCGATGG-3’
	R	5’- CTTCCTCTGCAGTTTTTCCA-3’
***mtl-1***	F	5’-GGAGGCCAGTGAGAAAAA ATG-3’
	R	5’-GCTTCTGCTCTGCACAATGAC-3’
***hsp-12.6***	F	5’-TGGCCACTTCAAAAGGGAG-3’
	R	5’-CTCTTTTGGGAGGAAGTTATGG-3’
***hsp-16.1***	F	5’- CCACTATTTCCGTCCAGCTC-3’
	R	5’- TGGAGAGCCTCTGCAAACTG-3’
***hsp-16.2***	F	5’- TATGGCTCTGATGGAACG -3’
	R	5’- GATTGATAGCGTACGACC -3’
***dod-3***	F	5’- GGAGTCCTGCTCTCAGATGAA-3’
	R	5’- ACATGAACACCGGCTCATTC-3’
***act-1***	F	5'- CCAGGAATTGCTGATCGTATGCAGAA-3’
	R	5'- TGGAGAGGGAAGCGAGGATAGA-3’

F: Forward Prime; R: Reverse Primer.

### Sulforaphane mediates longevity via DAF-2/DAF-16 signaling

**Figure 7:**
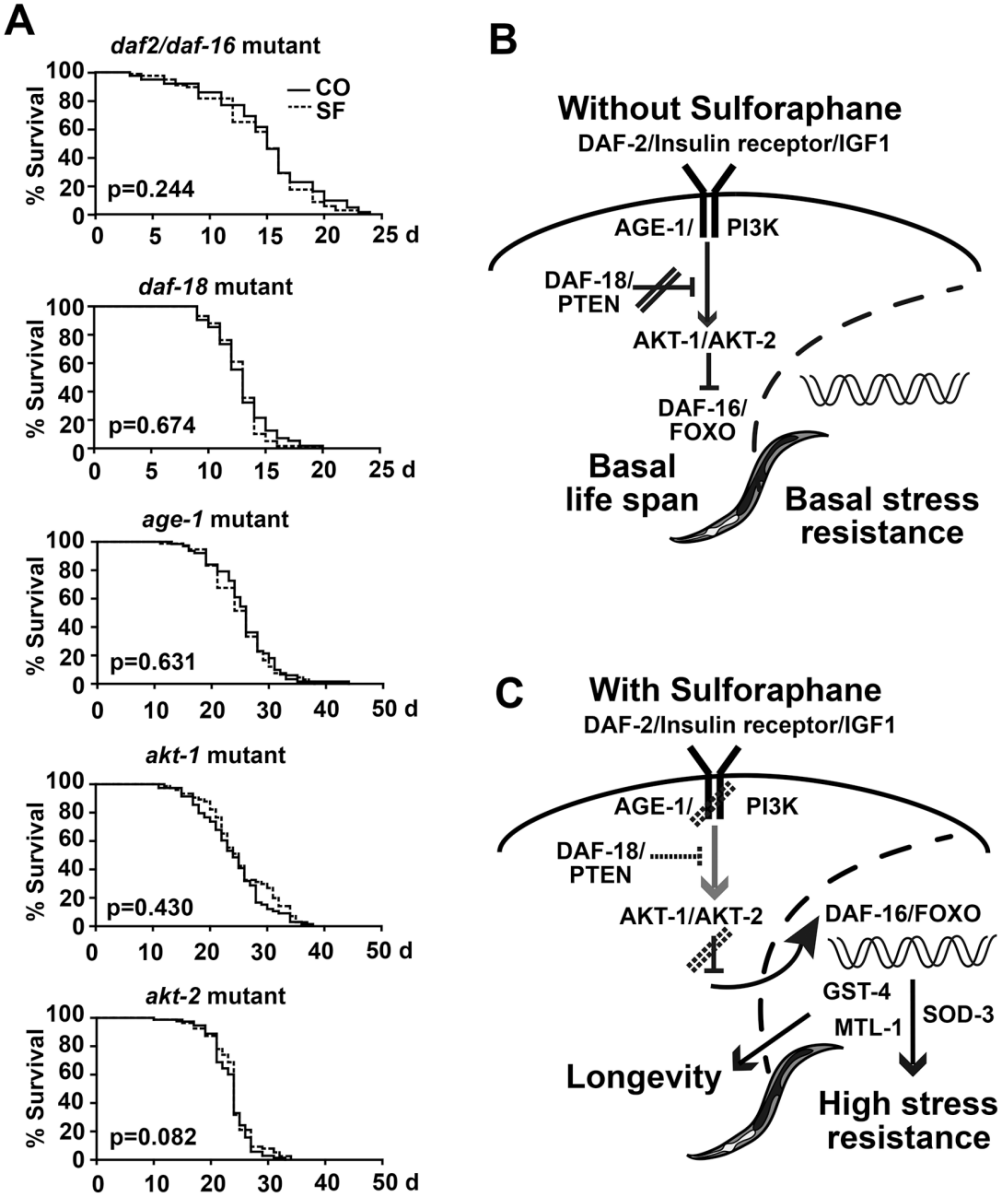
**Sulforaphane mediates longevity via DAF-2/DAF-16 signaling.** (**A**) Kaplan-Meier survival curves were generated by the use of *C. elegans* strains with mutations in *daf-2/daf-16, daf-18, age-1, akt-1* and *akt-2, and the P values, indicating significance or not, are given within the diagrams.* (**B**, **C**) Schematic representation of DAF-2/DAF-16 signaling under physiological conditions and after treatment with sulforaphane, as described in detail in the results section.

To clarify the sulforaphane-induced signaling chain between DAF-2 and DAF-16, we studied the proteins between them through the use of mutated *C. elegans* strains. We first fed *daf-2/daf-16* double mutant worms in the presence or absence of sulforaphane and performed a lifespan assay. Sulforaphane was not able to prolong their lifespan (Figure 7A, Tables 1, 2), unlike what was seen in the wild-type N2 *C. elegans* strain (compare Figure 1B). Similarly, sulforaphane did not prolong the lifespan of *C. elegans* strains with mutations in *daf-18*, *age-1*, *akt-1*, and *akt-2*. These results suggest that under basal physiological conditions, without sulforaphane, the DAF-2/insulin receptor/IGF1 pathway is active and signals via AKT1/AKT2, which prevents the nuclear translocation of DAF-16/FOXO [21] and, thus, the activation of life-prolonging target genes (Figure 7B). This scenario is blocked by DAF-18/PTEN, which enables the basal nuclear transcriptional activity of DAF-16/FOXO and, thus, the expression of genes necessary for a basal stress response and a normal life expectancy [39]. In the presence of sulforaphane, signaling by the DAF-2/insulin/IGF-1 receptor, AKT-1/AKT-2, and possibly DAF-18/PTEN is inhibited (Figure 7C). This activates the nuclear translocation of DAF-16/FOXO, in turn inducing the expression of *sod-3*, *mtl-1* and *gst-4*, which mediate stress resistance and longevity [22–25].

## DISCUSSION

Here, we examined whether the natural isothiocyanate sulforaphane, which is enriched in Brassicaceae vegetables, especially in broccoli, might exhibit anti-aging effects in *C. elegans*. We found that sulforaphane prolonged the lifespan as well as the healthspan of *C. elegans*. Through the use of several mutant *C. elegans* strains and different methods for the characterization of phenotypes, behavior and signaling cascades, we showed that the sulforaphane-mediated inhibition of DAF-2/insulin receptor/IGF-1 signaling and the activation of DAF-16/FOXO transcriptional nuclear activity are crucially involved in the sulforaphane-mediated lifespan prolongation of *C. elegans*.

Our report is the first to show that sulforaphane can extend the lifespan. Previous studies have demonstrated that sulforaphane is a chemopreventive agent against many types of cancer [40] and that it can act in synergy with chemotherapeutic drugs to increase chemosensitivity [41]. It was further known that vegetables rich in sulforaphane and other similar substances are beneficial for the reduction of inflammation and cancer risk, the stimulation of cellular antioxidant defense and digestion health [42]. Our study now confirms in *C. elegans* that low-dose sulforaphane is nontoxic and promotes longevity. This is consistent with the results of our recent pilot study in which patients with pancreatic cancer received broccoli sprouts in addition to palliative chemotherapy [43]. The patients in the broccoli sprout group survived longer than those in the placebo group, although these results were not statistically significant. This lack of significance may have been due to the small number of patients participating in the pilot study, and future larger studies must confirm these preliminary results.

In recent studies, life-extending drugs and other interventions have not necessarily promoted healthy aging [44]. However, with the increasing life expectancy of the global population, the significance of delaying aging seems to be far more important than extending lifespan. According to our results, sulforaphane not only increased the average lifespan by approximately 17% but also improved the healthspan, which is of great significance for the development of new anti-aging drugs. Our results showed that sulforaphane increased appetite, food intake and mobility and reduced the accumulation of lipofuscin and ROS. These phenotypic changes are related to healthy longevity [3] and thus suggest the consumption of broccoli or supplements obtained from this vegetable as a new promising anti-aging strategy.

Our data regarding the sulforaphane-induced stress resistance of *C. elegans* are related to the free radical theory of aging, which indicates that free radical damage to cells and DNA is the main cause of aging [45]. Oxidative stress caused by excessive levels of ROS is thought to be the main cause of aging-related diseases [46]. With increased age, the resistance of *C. elegans* to stress declines dramatically, and increased levels of ROS accelerate the aging process. Our findings regarding the sulforaphane-mediated inhibition of stress consequences induced by oxidative agents are consistent with previous data indicating that the inhibition of ROS levels causes increased resistance to oxidative stress and extends the lifespan of *C. elegans* [47]. In addition, our data reveal that sulforaphane does not enhance the resistance of *daf-2* mutants toward oxidative stress, indicating that insulin signaling is required for the sulforaphane-mediated protection from oxidative stress. However, we observed that sulforaphane does not prevent the consequences of heat stress, possibly because of the different signaling pathways that are involved in oxidative stress and heat stress [48]. It could be that sulforaphane can only regulate the signaling chains that cause oxidative stress. Alternatively, the heat shock temperature of 35° C that we used might have been too high or the exposure time too long, which could have caused a necrotic breakdown of cells.

According to our results, sulforaphane exerts its effects via DAF-2/DAF-16 insulin/IGF-1 signaling rather than through other pathways associated with longevity and healthspan, such as dietary restriction, mitochondrial respiration, or germline signaling [4]. We base this conclusion on our Kaplan-Meier survival experiments involving *C. elegans* mutant (mt) strains showing alterations in insulin signaling (mt daf-2), dietary restriction (mt eat-2), germline regulation (mt sir-2) and mitochondrial respiration (mt isp-1). Recent studies confirm our assumptions and show that several natural compounds utilize insulin signaling to delay aging [49, 50]. This may be because insulin/IGF-1 signaling plays an important role in development, aging, stress resistance and metabolism in a number of organisms [51].

In line with this assumption, we observed the sulforaphane-induced nuclear translocation of DAF-16/FOXO. This is in accord with the finding that DAF-16/FOXO is one of the major transcription factors required to achieve an extended lifespan, as observed in *C. elegans* strains with a mutation in the insulin-like DAF-2 receptor [23]. Consequently, in wild-type *C. elegans*, but not in the daf-16 mutant strain, we detected sulforaphane-induced increases in the expression of the DAF-16 target genes *sod-3*, *mtl-1* and *gst-4*, which are known to mediate stress resistance and longevity [22] [23–25]. In this regard, SOD-3 encodes an iron/manganese superoxide dismutase, that defends against oxidative stress and promotes normal lifespan [52]. GST-4 has a key role in the detoxification of oxidative stress products [52], and MTL-1 encodes metallothionein, which is a small cysteine-rich metal-binding protein, that has been implicated in longevity and proteotoxic stress [53]. In addition, the absence of a sulforaphane-induced increase in lifespan in mutants downstream of DAF-2 and upstream of DAF-16, including *mt age-1, mt daf-18*, *mt akt-1/2* (corresponding to the mammalian homologues PI3K, PTEN, and AKT, respectively) further confirms our assumption that sulforaphane prolongs the lifespan via DAF-2/DAF-16 signaling.

We did not find an effect of sulforaphane on the brood size of *C. elegans*. This confirms our hypothesis that germline signaling is not involved in sulforaphane-induced longevity.

In conclusion, we are the first to report that sulforaphane prolongs the lifespan and increases the healthspan of *C. elegans* through the inhibition of DAF-2/insulin/IGF-1 signaling and the activation of DAF-16/FOXO nuclear transcription in *C. elegans*. Our study provides a promising hint regarding the suitability of sulforaphane as a new anti-aging drug. However, additional studies in invertebrates and mammalian model organisms are necessary to expand our findings.

## MATERIALS AND METHODS

### *C. elegans* strains

The *C. elegans* strains N2/Bristol (wildtype reference strain) [54], CB1370 *daf-2(e1370) III* [30], DA1116 *eat-2(ad1116) II* [31, 32], VC199 *sir-2.1(ok434) IV* [33], MQ887 *isp-1(qm150) IV* [34, 35], CF1038 *daf-16(mu86) I* [36], GR1309 *daf-16(m26) I* [36]*;* RB712 *daf-18(ok480) IV [55, 56],* RB759 *akt-1(ok525) V* [57], VC204 *akt-2(ok393) X* [57, 58]and the reporter strain TJ356 *daf-16(zls356) IV* [37, 38], CL2166 *dvIs19 [(pAF15)gst-4p::GFP::NLS] III*, CF1553 *muIs84 [(pAD76) sod-3p::GFP + rol-6(su1006)]* were used (Table 1). All worm strains were obtained from the Caenorhabditis Genetics Center (CGC, University of Minnesota, Minneapolis, MN, USA).

### Culture and sampling of *C. elegans*

*C. elegans* strains were cultured in NGM nematode growth medium (0.3% NaCl, 1.7% agar, 0.3% peptone, 5 mg/ml cholesterol, 1 M KPO_4_, 1 M MgSO_4_) seeded with the *E. coli* OP50 strain (CGC, University of Minnesota, Minneapolis, MN, USA), which served as the major food source. The incubation temperature was 20° C, except for the CB1370 *daf-2 (e1370)III* strain, which was cultured at 15° C (Table 1). The worms were maintained according to the standard protocol as previously described [59].

### Adaptation of sulforaphane concentrations to bacterial growth

To prevent the OP50 food bacteria from being killed by sulforaphane, which would lead to the starvation of *C. elegans* and, thus, to false survival curves, we determined the maximum sulforaphane concentration tolerated by OP50. We used the broth microdilution method, which was originally intended for the determination of the susceptibility of a given microorganism to antibiotics [60]. OP50 cells were cultured overnight and diluted to an OD_600_ of ~0.1. Then, 0.1 ml of the bacterial solution was pipetted into single wells of a 96-well plate containing sulforaphane at concentrations of 8 μM to 1000 μM, followed by culture at 37° C for 48 h. The specific sulforaphane concentration at which the bacteria stopped growing was defined as the minimum inhibitory concentration. Next, we determined the most suitable sulforaphane concentration according to the OP50 growth curve. For this purpose, we added 15 ml of the bacterial solution (OD_600_ of ~0.1) to 50 ml Falcon tubes containing sulforaphane at concentrations of 100 μM to 400 μM, followed by shaking at 37° C at 200 rpm for 8 h. The OD_600_ was measured in 30-min intervals through the use of a Helios Delta spectrophotometer (Thermo Spectronic, London, United Kingdom) during a period of 8 h, as previously described [29]. The growth curve was created using GraphPad Prism 6.0 software (GraphPad Software Inc., California, USA).

### Lifespan assay

In lifespan assays, *C. elegans* was incubated in NGM with or without sulforaphane, and lifespan assays were performed as previously described [61]. In short, thirty 4-day-old worms were transferred to NGM plates and allowed to lay eggs for 3 h. Then, the nematodes were removed. Five hours later—with small variations depending on the worm strain—larvae at stage 1 (L1) hatched. After the development of late L4 larvae approximately 2 days later, the worms were transferred to fresh NGM plates. This time point was considered day 0 of the lifespan assay. During the reproductive period, the worms were transferred daily to freshly prepared plates to separate the adults from their progeny; thereafter, at ~day 10, transfer was carried out every second day. Worms that no longer responded to a gentle touch with the picking wire were scored as dead and excluded from the plates. The end point was considered to be the day when all worms were dead. Worms showing internal hatching and missing worms were censored. The Kaplan-Meier method was used to calculate the percentage of survival and the mean lifespan by using SPSS 22.0 software, and all lifespan results are summarized in Table 2.

### Bacterial avoidance assay

To evaluate the preference of *C. elegans* for sulforaphane, OP50 alone and OP50 mixed with 100 μM sulforaphane were seeded on opposite sides of an NGM plate, and 50 worms were placed in the center of the plate. Then, the number of worms on each side was counted after 3 h and 6 h.

### mRNA extraction and real-time PCR

Approximately 200 synchronized L4 larvae were transferred to fresh NGM plates with or without sulforaphane. After 3 days, adult worms were collected and washed 3× with M9 buffer (3 g KH_2_PO_4_, 6 g Na_2_HPO_4_, 5 g NaCl, 1 ml 1 M MgSO_4_, in 1 L H_2_O_bidest_) [54]. The RNeasy Mini Kit (QIAGEN, Manheim, Germany) was used for the extraction of total RNA according to the instructions of the manufacturer. Real-time PCR was performed by using the SYBR Green PCR Master Mix kit (Thermo Scientific, Schwerte, Germany). The PCR primer sequences were designed based on reference to previous studies [62–64]. The PCR pairs were synthesized by Eurofins Scientific (Mannheim, Germany), and the sequences are given in Table 3.

### Quantitation of GST-4::GFP and SOD-3::GFP expression

The reporter strains CF1553 and CL2166 were used to visualize SOD-3 and GST-4 expression, respectively. Age-synchronized L4 larvae were incubated in NGM agar plates containing 100 μM sulforaphane and *E. coli* OP50. After 3 days treatment, worms were placed on a 2% agarose pad in a drop of PBS containing 10 μM of the paralyzing agent levamisole. Then, the expression of SOD-3 and GST-4 was measured by quantifying the fluorescence of the reporter protein GFP. The intensity of fluorescence was analyzed using ImageJ (National Institutes of Health, Bethesda, MD, USA).

### Detection of intracellular ROS

Fifty 10-day-old worms, which were grown in the presence or absence of sulforaphane, were washed 3× with M9 buffer. The worms were then incubated in 5 μM fluorescent dihydroethidium (DHE, Thermo Scientific, Schwerte, Germany) in M9 buffer in the dark at 20° C for 1 h. DHE is taken up by the worms and oxidized by superoxide to form 2-hydroxyethidium (2-EOH). The worms were washed once with M9 buffer to remove excess dye and then mounted on a glass slide with a drop of 10 mM sodium azide (AppliChem GmbH, Darmstadt, Germany) for paralysis. Total fluorescence was analyzed with a Leica DMRB fluorescence microscope (Leica, Wetzlar, Germany), pictures were taken with a Zeiss camera (Carl Zeiss, Oberkochen, Germany), and the fluorescence intensity was analyzed by using ImageJ (National Institutes of Health, Bethesda, MD, USA). Likewise, fifty 10-day-old worms were washed 3× with M9 buffer, followed by protein extraction with RIPA buffer. The fluorescent dye CM-H_2_DCFDA (Sigma, Munich, Germany) at a concentration of 20 μM was incubated with 50 μL of the lysate for 30 min at 20° C in the dark. CM-H_2_CFDA is oxidized by ROS into the fluorescent dye DCF, which was quantified through the use of a microplate reader (BMG LABTECH, Ortenberg, Germany) at an excitation wavelength of 485 nm and an emission wavelength of 520 nm.

### Nuclear localization of DAF-16

The *C. elegans* reporter strain TJ356, which expresses a DAF-16-GFP fusion protein [37, 38], was treated with sulforaphane for three days, starting with L4 larvae. Then, the worms were transferred to a 2% agarose pad on a glass slide. The worms were anesthetized by adding one drop (~20 μl) of 10 μM levamisole (Sigma-Aldrich, Muenchen, Germany) to the agarose pad. The expression of GFP was examined by fluorescence microscopy. To exclude interference with endogenous fluorescence, it was ensured that the worms had consumed sufficient food prior to the experiment. Specifically, the picked worms were transferred to a new food plate and allowed to eat for one hour. For the analysis and scoring of the nuclear localization of DAF-16, the worms were divided into three groups according the intensity of fluorescence expression: low, intermediate, and high. If the green fluorescent nuclei in a worm accounted for less than 50% of the total nuclei, the fluorescence expression was considered low. Similarly, the number of green fluorescent nuclei in a worm was close to or greater than 50% of the total number of nuclei, and nuclear transcription was considered intermediate or high. The percentage of DAF-16 nuclear localization in each group is reported as the ratio of the value for each group to the total.

### Oxidative and heat stress assay

The oxidative stress assay and the heat stress assay were performed as described previously [61, 65]. In brief, synchronized L4 larvae were treated with sulforaphane. After 48 h, the worms of each group (each group contained 100 worms in total) were transferred to liquid NGM medium and exposed to a 120 μM concentration of the pro-oxidant juglone (5-hydroxy-1,4-naphthoquinone, Sigma-Aldrich GmbH, Steinheim, Germany) in a 12-well plate for 24 h. Dead and living worms were subsequently counted. For the heat stress assay, worms were treated for 2 days with sulforaphane and then incubated at 35° C for 8 h or 12 h before analysis. The worms were considered dead when they did not respond to a gentle touch with the picking wire.

### Pharyngeal pumping assay

This assay was performed as previously described [66]. In short, L4 larvae were treated with sulforaphane or were left untreated in the control group. The pumping frequency of the terminal pharyngeal bulb of each worm was counted for 60 seconds at days 6, 9 and 12 after treatment. During this time, the adult worms were picked daily and reseeded on a new NGF plate to avoid the development of offspring. An inverted microscope (Leica, Wetzlar, Germany) was used to measure the pumping rate in at least 20 worms from each group. In addition, a differential interference contrast microscope (Ni-E, Nikon, Germany) was used to photograph the pharyngeal structure of *C. elegans*.

### Mobility/body bending assay

This worm mobility behavior assessment was used to reflect the functional state of motor neurons and muscle cells [67, 68]. Body bends were used to reflect worm mobility behavior. After sulforaphane exposure of the worms at days 6, 9 and 12, the single worms were transferred to freshly made NGM plates without food. A change in the posterior bulb direction or regular swinging of the head was considered to represent a body bend. For each treatment, twenty worms were examined. Body bending was counted for 60 seconds as body bends/min.

### Brood size assay

For the brood size assay, synchronized L4 larvae were isolated and seeded on NGM agar plates, with a total of 12 worms per group. Sulforaphane was added to the NGM agar plates, or the plates were left untreated in the control group, followed by incubation for 24 h at 20° C. The adult worms were transferred to fresh NGM plates daily to separate them from their offspring. Eggs were counted daily under a microscope for a period of 7 days to obtain the brood size. The internal structure of the pregnant hermaphrodite was assessed with a differential interference contrast microscope (Ni-E, Nikon, Germany).

### Lipofuscin assay

The accumulation of the aging, or "wear-and-tear", pigment lipofuscin was assessed as described previously [69]. L4 larvae were incubated with sulforaphane or were left untreated. In 12-day-old adult worms, the autofluorescence of intestinal lipofuscin was measured through the use of blue excitation light (405-488 nm) and fluorescence microscopy. The fluorescence intensity of 20 worms was quantified using ImageJ software to determine lipofuscin levels.

### Statistical analysis

Lifespan assays were repeated at least three times unless otherwise stated. The results of the lifespan assays and the mean lifespan were analyzed with the Kaplan-Meier method, and the p-values of survival differences were determined with the log-rank test. Differences between two groups were assessed using Student’s t-test. SPSS 22.0 and GraphPad Prism 6 were used for all other statistical analyses. P-values <0.05 were considered to be significant.

## Supplementary Material

Supplementary Figure 1
