# Supplementary material for: Sulforaphane promotes C. elegans longevity and healthspan via DAF-16/DAF-2 insulin/IGF-1 signaling
Source: Aging (Albany NY). 2021 Jan 20;13(2):1649–70. doi: 10.18632/aging.202512 (PMC7880325; doi:10.18632/aging.202512)
Supplement: Supplementary Figure 1 [file aging-13-202512-s001.pdf]

SUPPLEMENTARY FIGURE

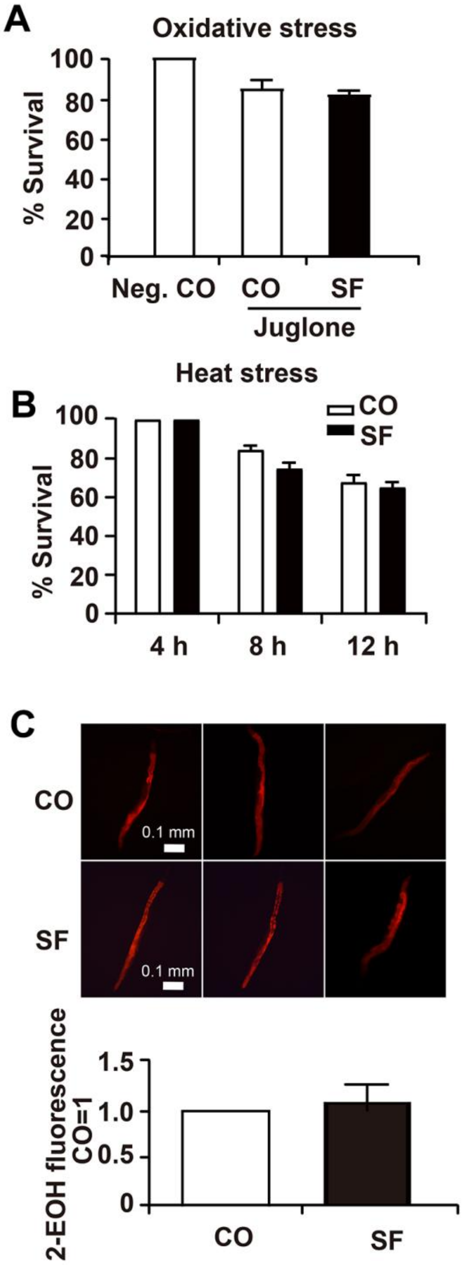

Supplementary Figure 1. Sulforaphane does not influence stress resistance following daf-2 mutation. (**A**) Daf-2 mutant C. elegans worms were prepared, treated and analyzed as described in Figure 4A. (**B**) Likewise, daf-2 mutant worms were evaluated as described in Fig. 4B, or (**C**) as described in Figure 4C. Representative images at 100× magnification are shown, and the scale bar indicates 0.1 mm. The data are expressed as the means ±SD. *P<0.05.
